# Effect of age on the outcome of renal transplantation: A single-center experience

**DOI:** 10.12669/pjms.324.10094

**Published:** 2016

**Authors:** Faruk Ozkul, Halil Erbis, Vural Taner Yilmaz, Huseyin Kocak, Ibrahim Ali Osmanoglu, Ayhan Dinckan

**Affiliations:** 1 Faruk Ozkul, Department of General Surgery, School of Medicine, Canakkale Onsekiz Mart University, Canakkale, Turkey; 2 Halil Erbis, Department of General Surgery, School of Medicine, Akdeniz University, Antalya, Turkey; 3 Vural Taner Yilmaz, Department of Internal Medicine, Division of Nephrology, School of Medicine, Akdeniz University, Antalya, Turkey; 4 Huseyin Kocak, Department of Internal Medicine, Division of Nephrology, School of Medicine, Akdeniz University, Antalya, Turkey; 5 Ibrahim Ali Osmanoglu, Department of Internal Medicine, Division of Nephrology, School of Medicine, Akdeniz University, Antalya, Turkey; 6 Ayhan Dinckan, Department of General Surgery, School of Medicine, Akdeniz University, Antalya, Turkey

**Keywords:** Age, Renal transplantation Survival

## Abstract

**Objectives:**

To analyze the effects of old age on renal transplantation (Tx) results and graft survival, and compared elderly patient population with the young patients.

**Methods:**

A total of 1946 renal transplant were performed from 1537 living and 409 cadaveric donors between 2003 and 2014. The recipients were divided into two groups according to their age at the time of transplantation. The young age group consisted of 18-59-year-old, and the elderly group consisted of the ones ≥ 60 years.

**Results:**

Acute rejection was seen in 19.5% of the young age group while this rate was 16.7% in the old age group (p=0.535). DGF was seen in 6.3% of the young age group, and in 13.5% of the old age group (p<0.001). Analysis of the overall survival rates demonstrated that 1.6% of the patients in the young age group and 6.8% of the patients in the old age groups died (p=0.003).

**Conclusions:**

Renal transplant had high graft survival rates in the elderly as in the young patients. However, the risks for complications were higher in the older age group compared to the younger age group. Thus, it is important to make a careful selection among elderly candidates for renal transplantation.

## INTRODUCTION

The number of the elderly patients with end-stage kidney disease who are candidates for renal transplantation (Tx) has been increasing as the world population gets older. In addition, elderly people have been increasingly act as donors owing to limited number of living and cadaver organ donors. Currently, old age has not been accepted as a contraindication for renal transplant. However, short life expectancy as well as higher rejection and medical complication rates related to comorbidities cause a bias for renal Tx in the elderly patients.

Various centers in the developed countries reported the results of renal transplant in the elderly in order to analyze the risks of elderly kidney donors and recipients better, and to determine the effects of age on graft survival.^1^-^5^ Although a clear consensus has not been reached, it has been currently supposed that renal Tx could be employed safely in selected elderly patients. However, we suppose that renal transplant results in the elderly patients should be analyzed before reaching this conclusion.

For this purpose, in this large single-center study, we analyzed the effects of old age on renal Tx results and graft survival, and compared elderly patient population with the young patients.

## METHODS

### Subjects

A total of 1946 renal transplant were performed from 1537 living and 409 cadaveric donors between January 2003 and June 2014 in our center. The recipients and living donors were divided into two groups according to their age at the time of transplantation. The young age group consisted of 18-59-year-old participants, and the elderly group consisted of the ones ≥ 60 years of age. The exclusion criteria were the ones that had another organ transplant besides the kidney, and a pediatric age. The immunosuppression protocols were similar in two age groups.

We retrospectively reviewed the medical data of the donors including age, gender, body mass index (BMI), comorbidities, blood pressure, kidney size, serum creatinine level, and glomerular filtration rate (GFR). We also reviewed the data of the recipients regarding age, gender, primary renal disease, mean blood pressure, serum creatinine level, duration of dialysis, history of hypertension and/or diabetes, HLA haplotype matches, vascular anastomosis time, total surgical time, and surgical complications.

The mean follow-up period was 120.7±24 months with a minimum period of 12 months. The data were collected 1, 6, and 12 months after transplantation, and included presence of delayed graft function (DGF), acute rejection episodes, total hospital stay, and the laboratory parameters. The primary endpoints were patient and graft survival. Graft failure was defined as return to dialysis. Death of the recipient was considered as the patient loss. DGF was defined as the need for dialysis within the first week after Tx. Donor follow-up data were also collected.

### Statistical analysis

The statistical analyses were performed using SPSS for Windows, version 19.0. Chi-square test was used for univariate analysis of the categorical variables, and Mann-Whitney U test was used for the continuous variables. Graft and patient survivals were calculated with the Kaplan-Meier method. Logistic regression analysis was used to identify the risk factors associated with graft survival and patient outcomes. A p value smaller than 0.05 was considered as statistically significant.

## RESULTS

Among 1946 Tx recipients, 1859 patients (95.5%) were younger than 60 years of age, and 87 patients (4.5%) were ≥ 60 years of age. The mean age of elderly group was 63.7±3.6 years, compared with 36.9±11.3 years in the younger group. Seventy percent of the patients were males in the elderly group versus 67.3% in the younger group (p=0.590). The mean age of the donor group was 44.2±12.5 (18 – 87) years. There were 1719 (88.3%) patients in 18 – 59 age group, and 227 (11.7%) patients ≥ 60 years of age, and 48.6% of them were males and 51.4% were females.

**Fig.1:**
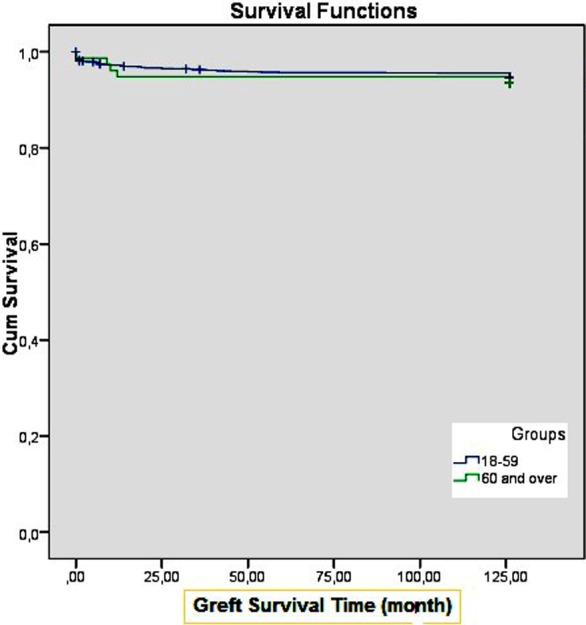
Survival Functions.

There was no difference between young and old age groups for hospital stay (8.7±6 vs 9.3±4.2 days, p=0.053). Acute rejection was seen in 19.5% of the young age group while this rate was 16.7% in the old age group (p=0.535). DGF was seen in 6.3% of the young age group, and in 13.5% of the old age group (p<0.001). The creatinine levels measured 1, 6, and 12 months after Tx were similar in the young and old age groups (p=0.417, p=0.231, p=0.322; respectively). There was no difference between the young and old recipient age groups for graft survival rate, after a mean follow up period of 120 months (p=0.972; Fig.1).

Analysis of the overall survival rates demonstrated that 1.6% of the patients in the young age group and 6.8% of the patients in the old age groups died (p=0.003). The most frequent causes of death were cardiovascular diseases (32.3%), infection (29%) and cranial bleeding (12.9%) in the young age group, however the most frequent causes of death were infection (60%), cardiovascular diseases (20%) and liver deficiency (20%) in the old age group. The demographic data and the results obtained after Tx in young and old age groups are summarized in Table-I.

**Table-I:** Patient characteristics and postoperative data.

	Age < 60 years	Age ≥ 60 years	p value
No. patients (%)	1859	87 (4.5%)	
Mean recepient age ± SD, years	36.9±11.3 years	63.7±3.6 years	
Recepient gender			
Male / Female (%)	1252/607 (67.3%)	61/26 (70%)	0.590
Dialysis pretransplant (%)	82.4%	85.7%	0.748
Mean cold ischemia time (h)	3.2±5.7	3.7±5.7	0.687
Mean ± SD hospitalization time (days)	8.7±6 days	9.3±4.2	0.053
Mean ± SD serum creatinine 1 yr (mg/dL)	1.2±0.6	1.3±0.7	0.322
Acute rejection rate (%)	19.5%	16.7%	0.535
Delayed graft function rate (%)	6.3%	6.8%	<0.001*
Graft survival rate (%)	94.7%	92.5%	0.635
Death rate (%)	1.6%	6.8%	0.003*

* Significant at 0.05 level.

## DISCUSSION

The results of our study indicated a smaller survival rate and a higher DGF prevalence in the recipients ≥ 60 years of age when compared to younger recipients, however two groups were similar for graft survival. Those results are in accordance with the results of the previous studies that compared old and young recipients and concluded that those groups were not different for graft survival.^1^-^9^

In a recent study, Bronzatto et al. reported that an older donor age, history of systemic hypertension and a higher BMI were associated with DGF.^10^ In another study, Bardonnaud et al.^11^ showed that DGF was associated with a longer hospital stay, the complications related to a longer hospital stay and a higher health care cost. We found a significantly higher DGF rate in the older group when compared to the younger group. However, we did not find any differences between the older and younger groups for hospital stay, and related complications.

The effect of the age of the recipient on renal Tx outcomes remains controversial. The results of this study suggested that the age of the recipient did not affect graft survival. Older and younger age groups were similar for kidney functions in the long term. On top of that, acute rejection rate was lower in the older age groups when compared to younger age group, although this result did not reach statistical significance. It has been assumed that aging is associated with a progressive decline in the immune functions.^12^ This may explain the reason for the higher acute rejection rate in the younger age group.

Analysis of the causes of mortality revealed cardiovascular diseases as the most frequent cause of death in the younger recipients; however mortality was most frequently resulted from infection in the older recipients. A number of centers have reported cardiovascular diseases as the most important cause of post- transplant mortality. However, Ossareh et al.^1^ reported mortality as the most important cause of infection in both young and old age groups. Mortality due to infection or cardiovascular diseases may be minimized with a good pre-Tx evaluation and the measures taken during the hospitalization. Therefore, every transplant center should analyze its results, and take necessary precautions. All of our patients underwent an extensive preoperative evaluation for cardiac and infectious risks before transplantation, and this may explain higher allograft and patient survival rates in our study.

### Limitation of the study

Our study has some limitations. The most important one is retrospective nature of our study. Therefore, further prospective studies are needed to confirm our results. We suppose that the results of this single-center study that analyzed the results of renal Tx in the elderly in a developing country would contribute significantly to the literature.

## CONCLUSION

Our results indicated that renal Tx had high graft survival rates in the elderly as in the young patients. However, the risks for complications and mortality rate were higher in the older age group compared to the younger age group. Thus, it is important to make a careful selection among elderly candidates for renal transplant.^9^
